# Effectiveness of self-managed abortion during the COVID-19 pandemic: Results from a pooled analysis of two prospective, observational cohort studies in Nigeria

**DOI:** 10.1371/journal.pgph.0001139

**Published:** 2022-10-20

**Authors:** Ijeoma Egwuatu, Sybil Nmezi, Ruvani Jayaweera, Relebohile Motana, Belén Grosso, Ika Ayu Kristianingrum, Ruth Zurbriggen, Chiara Bercu, Caitlin Gerdts, Heidi Moseson

**Affiliations:** 1 Generation Initiative for Women and Youth, Lagos, Lagos State, Nigeria; 2 Ibis Reproductive Health, Oakland, California, United States of America; 3 Ibis Reproductive Health, Houghton, Johannesburg, South Africa; 4 La Revuelta Colectiva Feminista, Neuquén, Argentina; 5 Samsara, Java, Indonesia; Jhpiego, UNITED STATES

## Abstract

Globally, restrictions imposed by the COVID-19 pandemic altered access to clinical abortion care, as well as people’s ability to access abortion medications on their own. When clinical care is inaccessible, or when self-care is preferred, people use pills on their own, without clinical supervision, to end their pregnancies–a practice known as “self-managed” abortion. Little is known about experiences of self-managed abortion during the COVID-19 pandemic. The aim of this study was to measure experiences of self-managed abortion, including abortion completion, prior to and during the COVID-19 pandemic in Nigeria. Between October 2019—September 2020, we recruited callers to a safe abortion accompaniment group that provides information on self-managed abortion in Nigeria. Participants completed a baseline phone survey, and two follow-up phone surveys. Primary outcomes included burdens experienced prior to versus during the pandemic, and abortion completion. We calculated frequencies and percentages overall and by time period and compared outcomes across time periods using t-tests, Chi-squared tests, tests of proportion, and Mantel-Haenszel adjusted odds ratios. Overall, 807 participants were included in these analyses. Participants enrolled during the COVID-19 pandemic were more likely to report needing to borrow money (47.9% vs 29.4%) and find lodging outside the home (15.5% vs 3.2%) for their self-managed abortion than were those enrolled prior to the pandemic. Participants reported COVID-19 related difficulties most frequently during the earliest and strictest period of the lockdowns, particularly in obtaining and taking pills (32.4%), and comfort seeking healthcare (12.2%). Nearly all participants (95%) reported a complete abortion at last follow-up. Results from this study underscore the challenges Nigerians faced during the COVID-19 pandemic in self-managing their abortions, and also the essential role that a safe abortion hotline played in expanding access to safe abortion during a time when the formal healthcare system was less accessible and higher-risk.

## Introduction

The first case of the SARS-COV-2 virus (COVID-19), was reported in Nigeria in February 2020 [1]. Over the following months, the Nigerian Ministry of the Interior imposed a series of restrictions on movement and public gatherings in an effort to reduce the spread of the virus [2]. These lockdown measures limited people’s ability to access essential sexual and reproductive health care, including contraceptive methods, pregnancy tests, as well as services such as abortion at nearby clinics and health centers [3, 4]. Health centers across the country reported a 30–50% reduction in service utilization for family planning and related sexual and reproductive health services as compared to the pre-pandemic period [4], and over 25% of public health centers reported stock-outs of contraceptive products during phases of the COVID-19 related lockdowns [4].

Taken together, the impact of the COVID-19 pandemic and related restrictions are thought to have increased the incidence of unintended pregnancies worldwide, as well as changed the calculus as to whether a pregnancy is wanted or not due to life circumstances–and thereby increased the need for safe abortion care [3]. Despite the fact that Nigeria’s criminal and penal code allow abortion only when necessary to save the pregnant person’s life [5], abortion is common [6, 7]. Only 12% of women of reproductive age in Nigeria use any modern method of contraception [8], and approximately one in four pregnancies are thought to be unintended [7].

However, inequities exist in who is able to access safe versus unsafe methods of abortion [6, 9]. As a result of limited access to clinical abortion care and a desire for privacy and autonomy, some Nigerians choose to obtain and use medication abortion pills (misoprostol alone, or in combination with mifepristone) to end their pregnancies on their own, without the support of a clinician [9–14]. This practice of ending a pregnancy on one’s own is referred to as self-managed abortion [15]. Estimates suggest that more than half of the abortions in Nigeria each year are self-managed in a non-clinical setting [13]. A growing evidence base suggests that self-managed abortion is safe, effective, and high quality [15] including several recent studies in Nigeria [9–14, 16–18]. Recognizing the need for safe, self-managed abortions, grassroots feminist organizations have formed over several decades to counsel people with information and support through a self-managed medication abortion at no cost. These groups are referred to as safe abortion hotlines, or accompaniment groups. The model of care provided by these groups represents a powerful alternative to traditional clinic-based abortion care that potentially expanded access to safe abortion care for people during the COVID-19 pandemic [12].

However, there is a lack of data exploring how the COVID-19 pandemic has affected people’s self-managed abortion experiences, the potential barriers they faced, and how accompaniment groups facilitated access to self-managed abortion information and support during this ongoing global public health crisis. To address this gap, we conducted an analysis as part of a larger study to understand how the burdens and barriers faced by people self-managing an abortion with accompaniment support shifted over the course of the COVID-19 pandemic in Nigeria.

## Materials and methods

### Ethics statement

Ethical review for this research was completed by the Allendale Investigational Review Board in the United States and the National Health Research Ethics Committee in Nigeria. Study staff obtained verbal informed consent from all participants 18 years and older, and verbal informed assent from all participants 17 years and younger. Due to concerns about participant privacy and anonymity, the IRB and ethics committee waived the need for parental consent for minors. Funders of the study had no role in the study design, data collection, analysis, interpretation, writing of the report, or the decision to submit the paper for publication.

This analysis pooled data collected in two separate studies: (1) the Studying Accompaniment Feasibility and Effectiveness (SAFE) study [16, 19] and (2) the Abortion Service Quality (ASQ) study [17]; both are prospective, observational cohort studies that enrolled people who contacted safe abortion hotlines/accompaniment groups for information and support related to self-managed abortion. Researchers followed participants for approximately four weeks to assess pregnancy and quality of care outcomes; the primary outcomes for the SAFE study were self-managed abortion safety and effectiveness, and the primary outcomes for the ASQ study focused on quality of care; main outcomes from these studies have been published elsewhere [16, 17]. While the SAFE and ASQ studies collected data in several countries, this analysis presents results from Nigeria only.

Between October 2019 and September 2020 we recruited callers to a safe abortion hotline in Lagos, Nigeria. When a person contacted the safe abortion hotline seeking support with ending a pregnancy, counselors at the hotline helped the person verify the duration of the pregnancy, rule out contraindications for using the medications, and then provided World Health Organization-endorsed information on how to take pills to safely end a pregnancy, provided emotional and informational support on pain and symptom management, as well as if, when, and where to seek medical care if needed. Anyone who contacted the group during the study period requesting information about abortion for their own pregnancy was counted in a call log, and then screened for eligibility by study staff. Anyone ≥ 13 years of age (SAFE study) or ≥15 years of age (ASQ study), starting a new medication abortion process, with no contraindications to medication abortion [20], with a pregnancy up to 12 weeks was eligible to participate [19]. We excluded those experiencing ongoing symptoms (bleeding, cramping) of abortion or miscarriage, experiencing symptoms suggestive of ectopic pregnancy (low back pain, pain on side of the abdomen/pelvis, shoulder pain, dizziness/fainting); or anyone who did not want to be contacted by study staff.

At enrollment, hotline counselors administered a baseline questionnaire over the phone at the end of the first call with the caller. Subsequently, trained study coordinators contacted all participants at two additional time points (for SAFE:~7 days and ~21 days after taking the first medication abortion pills, for ASQ: ~7 days and ~30 days) to complete additional telephone surveys. Participants were provided between $10–25 USD in phone credit depending on how many follow-up surveys they completed.

The primary outcomes for this analysis included direct questions as to whether the COVID-19 pandemic and related lockdowns influenced participants ability to or comfort in various parts of the abortion process, including confirming their pregnancy, getting the pills, taking the pills, seeking medical care, and more. Specifically, questions followed this structure: “Did COVID-19 and the lockdowns affect your ability to confirm your pregnancy? (For example, to obtain a pregnancy test, to take a pregnancy test in privacy, to get an ultrasound, other.)” Additional COVID-19 related questions asked as to whether COVID-19 and related lockdowns influenced where participants had their abortion, the number of people who stayed in their home, and whether anyone found out about their abortion. Participants in both SAFE and ASQ studies answered these questions.

We also included three sets of secondary outcomes: the number of calls received by the hotline in each month, burdens experienced during the abortion process, and abortion completion. Burdens faced during the self-managed abortion process were only measured in the SAFE study, not in ASQ. To measure burdens, SAFE study coordinators asked participants “In preparing for, during, or in the weeks since you ended the pregnancy, did you have to do any of the following as part of the abortion process?” Answer choices included taking time off of work, forfeiting lost wages for time off from work, arranging childcare, finding lodging outside the home, borrowing money, selling something, traveling more than 30 minutes, or a participant-reported burden. Other variables measured in both studies included participant age and education, duration of pregnancy at enrolment, and medication regimen.

To measure abortion completion, both SAFE and ASQ studies utilized self-report. SAFE study participants self-reported abortion completion at the one and three week follow-ups, and ASQ study participants self-reported abortion completion at the one month follow-up. In the SAFE study, study coordinators asked participants two questions to ascertain abortion completion: “Do you feel that your abortion process is complete?”, as well as whether they reported receiving a manual vacuum aspiration or dilation and curettage procedure. In the ASQ study, study coordinators asked participants “Do you believe that you may still be pregnant today?” Participants who answered “yes” were classified as “not complete / not sure”, while those who responded with “no” were classified as “complete, inclusive of surgical intervention”.

For analysis, we compared frequencies and percentages for each outcome overall and across phases of the COVID-19 lockdown, and tested for differences in outcomes using t-tests, Chi-square tests, and tests of proportion. We also evaluated differences in the adjusted odds of each abortion-related burden by time period using the Mantel-Haenszel formula [21] to calculate stratum-specific odds ratios (ORs) for each education level (less than high school, high school graduate, and university graduate), adjusted for age (centered and continuous), and tested for homogeneity across stratum-specific estimates to evaluate for effect measure modification by education. Where no heterogeneity was found, we estimated a weighted average of the stratum-specific ORs using *mhodds* in Stata; where heterogeneity was found, we report adjusted ORs separately by strata of education to highlight effect measure modification [21]. We explored differences by age and education as these characteristics differed across pre versus during COVID-19 samples.

We defined COVID-19 pandemic related lockdowns in two ways: a binary formulation of “pre” versus “during” COVID-19, defined as any data gathered from anyone enrolled up to and including March 30, 2020 as “pre” (a five month period from October through March), and all data among those enrolled on March 31st and after as “during” (a six month period from March 31 through September). In an additional time category formulation, we defined COVID-19 related lockdowns in phases: Phase 1 from March 31-June 1, Phase 2 from June 2 –September 3, and Phase 3 from September 4 –December 31, 2020. These dates correspond with the dates that various movement and other pandemic related restrictions were enacted or lifted in Nigeria [22].

We analyzed data in STATA (version 15.1). While the ASQ and SAFE study samples sizes were powered to evaluate abortion completion and quality outcomes, not for the outcomes evaluated in this analysis, we calculated the minimum detectable absolute difference in proportions for a core outcome of this analysis, burdens faced prior to versus during the pandemic within the recruited sample sizes, and found the minimum detectable absolute difference to be 5.1% with 80% power and an alpha of 0.05.

## Results

**Fig 1:**
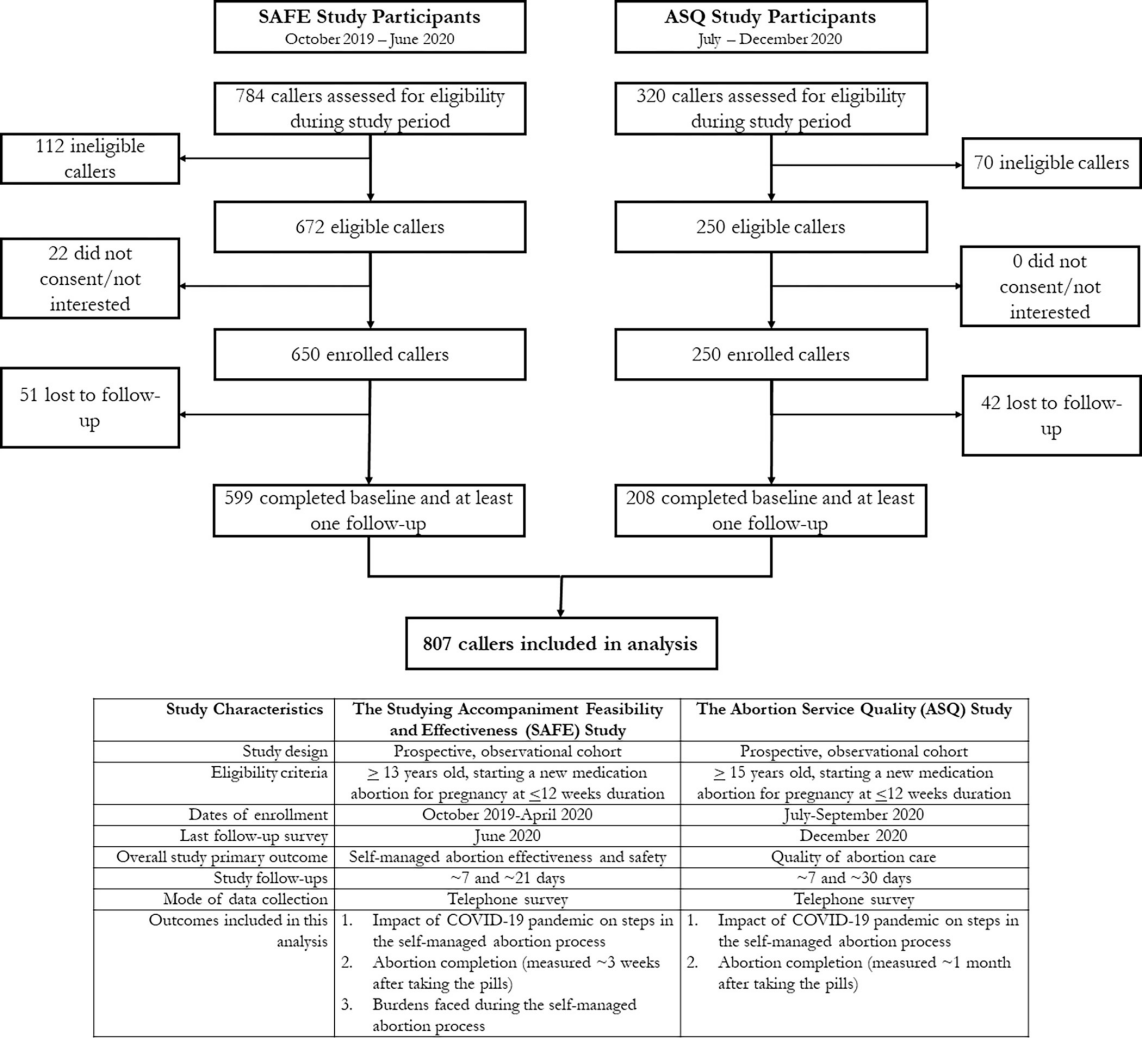
Screening and recruitment of callers to a safe abortion hotline in Nigeria for the ASQ and SAFE studies.

Across both studies, staff screened 1,104 callers for eligibility between October 2019 and September 2020, ranging from as few as 5 calls per month in December 2019, to as many as 333 calls per month in April 2020 during the strictest COVID-19 related lockdown phase. Among these 1,104 callers, 992 were eligible, and 900 enrolled (Fig 1). Of those enrolled, 807 (81% of those eligible) completed at least one follow-up and reported whether or not they obtained and took the medications. We include these 807 in these analyses; 411 (51%) enrolled prior to the COVID-19 pandemic, and 396 (49%) enrolled during the COVID-19 pandemic. These 807 participants include 396 people who were able to obtain wanted abortion care via a safe abortion hotline during the COVID-19 related lockdowns.

### Participant characteristics

Overall, most participants (63.1%) were between 20–29 years old, and over half completed post-secondary education (Table 1). At baseline, most participants (n = 468, 58%) were <7 weeks into their pregnancies; and most (n = 793, 98.3%) took misoprostol alone to end their pregnancies. Participants recruited after the onset of the COVID-19 pandemic were slightly younger and less well educated than were participants recruited prior to the onset of the pandemic.

**Table 1:** Participant sociodemographic and pregnancy characteristics among 807 callers to a safe abortion hotline in Nigeria between October 2019 and September 2020.

	Overall n = 807		Pre-COVID-19 Cohort^ n = 411		COVID-19 Cohort^^ n = 396		Ttest or Chi2 p-value
	n	%	n	%	n	%	
**Mean age (SD)**	27.9	(5.8)	29.1	(5.8)	26.6	(5.5)	**<0.001**
**Age Category**							**<0.001**
15-19y	27	3.3	11	2.7	16	4	
20-24y	217	26.9	77	18.7	140	35.4	
25-29y	292	36.2	149	36.3	143	36.1	
30-34y	140	17.3	86	20.9	54	13.6	
35-39y	103	12.8	71	17.3	32	8.1	
40-44y	23	2.9	14	3.4	9	2.3	
45-49y	4	0.5	2	0.5	2	0.5	
50y	1	0.1	1	0.2	0	0	
**Education**							**<0.001**
Less than high school grad	55	6.8	9	2.2	46	11.6	
High school grad	339	42	144	35	195	49.2	
University graduate	413	51.2	258	62.8	155	39.1	
**Duration of Pregnancy (Categories)**							0.22
< 7 weeks	468	58	253	61.6	215	54.3	
[7–9) weeks	222	27.5	103	25.1	119	30.1	
[9–12) weeks	96	11.9	45	10.9	51	12.9	
[12–22] weeks	21	2.6	10	2.4	11	2.8	
**Medication Abortion Regimen**							0.29
Mifepristone + Misoprostol	6	0.7	5	1.2	1	0.3	
Misoprostol alone	793	98.3	405	98.5	388	98	
No response/Unknown	8	0.9	1	0.2	7	1.8	** **

^ The Pre-COVID cohort includes participants recruited between Oct 19, 2019 and Mar 30, 2020.

^^ The COVID-19 cohort includes participants recruited between Mar 31, 2020 and Sept 30, 2020.

### Burdens experienced as part of the self-managed abortion process before and during COVID-19

Of all the burdens measured, participants most frequently reported having to borrow money as part of the abortion process and did so at a higher level during the COVID-19 pandemic as compared to before (Table 2). Similarly, participants were more likely to report having to find lodging outside of the home to self-manage their abortion during the pandemic than they were prior. After the onset of the pandemic, participants were less likely to report having to take time off from work or school than they were prior to the pandemic, and among participants with a university-level education or more, participants had lower odds of needing to travel more than 30 minutes as part of self-managing their abortion during the pandemic than they did prior to it.

**Table 2:** Burdens experienced as part of a self-managed abortion process among 599 callers to a safe abortion hotline in Nigeria between October 2019 and June 2020. The first three columns present unadjusted proportions overall and by time period, and the two right-most columns display Mantel-Haenzel odds ratios (OR) for each burden adjusted for participant age and education at baseline, with corresponding 95% Confidence Intervals for the OR. Due to an interaction detected with education, the ORs for COVID-19 pandemic and having to travel more than 30 minutes are presented stratified by educational level.

In preparing for, during, or in the weeks since you ended the pregnancy, did you have to do any of the following as part of the abortion process?	Overall		Pre-COVID-19 Cohort^		COVID-19 Cohort^^		Mantel-Haenzel Adjusted Odds Ratio	95%CI for OR_M-H_
	n = 599		n = 411		n = 188			
	n	%	n	%	n	%		
Borrow money	211	35.2	121	29.4	90	47.9	**1.79**	**(1.2, 2.7)**
Take time off of work/school	144	24	143	34.8	1	0.5	**0.01**	**(0.00, 0.1)**
Travel more than thirty minutes	128	21.4	92	22.4	36	19.1	** **	** **
*Participants with a secondary school level education vs < secondary*							1.27	(0.6, 2.5)
*Participants with a university level education vs < secondary*							**0.44**	**(0.2, 0.9)**
Arrange childcare	77	12.9	59	14.4	18	9.6	0.68	(0.4, 1.3)
Sell something	54	9	30	7.3	24	12.8	1.35	(0.7, 2.6)
Forfeit lost wages for time off	45	7.5	45	10.9	0	0	(collinear)	--
Find lodging outside of your home	42	7	13	3.2	29	15.4	**3.9**	**(1.7, 8.8)**
*Missing*	*43*	*7*.*2*	*43*	*10*.*5*	*0*	*0*	*--*	*--*
None of the above	139	23.2	87	21.2	52	27.7	1.1	(0.7, 1.7)

^ The Pre-COVID cohort includes participants recruited between Oct 19, 2019 and Mar 30, 2020.

^^ The COVID-19 cohort includes participants recruited and contributing data between Mar 31, 2020 and June 2020. This COVID-19 cohort covers a shorter time period than in other tables as this table includes responses only from participants in the SAFE study.

### Differences in the self-managed abortion experience across phases of the COVID-19 pandemic

Across the three specified phases of COVID-19 related lockdowns in Nigeria, participants reported the greatest difficulty accessing and taking pills in the second phase (Table 3). No substantial differences across phases were observed in where participants had their abortions or where they would have preferred to have their abortion–in all cases, the majority of participants had and preferred to have the abortion in a private room in their home. Across all other measured impacts of the pandemic, participants reported the greatest difficulties in the first and strictest phase of the lockdown. During phase I, participants most frequently reported a change in the number of people staying in the home, someone finding out about their abortion, changes in their ability to confirm their pregnancy, as well as their comfort or ability in seeking medical care during or after their abortion. Across all phases, participants reported lost wages as a direct result of the COVID-19 pandemic, most frequently in the first phase.

**Table 3:** Steps in the self-managed abortion process across phases of the COVID-19 pandemic reported by 396 callers to a safe abortion hotline in Nigeria between March and December 2020. A pre-COVID period is not included in this table as these questions were not asked prior to the COVID-19 pandemic.

	Overall		Lockdown phases implemented in Nigeria during the COVID-19 Pandemic						
			Phase 1: March 31-June 1		Phase 2: June 2-Sept 3		Phase 3: Sept 4—Dec 31		
	*n = 396*		*n = 188*		*n = 89*		*n = 119*		
	n	%	n	%	n	%	n	%	Chi2 P-value
**Thinking back to when you got the pills, would you describe your experience getting the pills as:**									
Very difficult	47	11.9	29	15.4	12	13.5	6	5	**0.003**
Somewhat difficult	104	26.3	42	22.3	33	37.1	29	24.4	
Somewhat easy	68	17.2	33	17.6	17	19.1	18	15.1	
Very easy	176	44.4	83	44.1	27	30.3	66	55.5	
Missing/not asked	1	0.3	1	0.5	0	0	0	0	
**Where did you do/have the abortion?**									0.11
In your home, in a private room	335	84.6	149	79.3	78	87.6	108	90.8	
In your home, not in a private room	3	0.8	3	1.6	0	0	0	0	
In a family member’s home	8	2.0	6	3.2	0	0	2	1.7	
In a friend’s home	44	11.1	27	14.4	8	9	9	7.6	
Somewhere else	4	1.0	3	1.6	1	1.1	0	0	
Missing/not asked	2	0.5	0	0	2	2.2	0	0	
**If the COVID-19 lock down were not in place, where would you have wanted to have the abortion?**									0.396
In your home, in a private room	347	87.6	158	84.0	80	89.9	109	91.6	
In your home, not in a private room	2	0.5	2	1.1	0	0	0	0	
In a family member’s home	5	1.3	3	1.6	0	0	2	1.7	
In a friend’s home	37	9.3	21	11.2	8	9	8	6.7	
Somewhere else	5	1.3	4	2.1	1	1.1	0	0	
**Has the COVID-19/lockdown changed the number of people who stay in your home?**									**<0.001**
Yes, more people	55	13.9	52	27.7	1	1.1	2	1.7	
Yes, less people	38	9.6	32	17.0	6	6.7	0	0	
No	303	76.5	104	55.3	82	92.1	117	98.3	
**Did anyone find out about your abortion as a result of the COVID-19 lockdown?**									**0.001**
Yes	12	3.0	12	6.4	0	0	0	0	
No	384	97.0	176	93.6	89	100	119	100	
**Did COVID-19/lockdown affect your ability to confirm your pregnancy?**									**0.001**
Yes	16	4.0	15	8.0	1	1.1	0	0	
No	380	96.0	173	92.0	88	98.9	119	100	
**Did COVID-19/lockdown affect your ability or comfort to GET/OBTAIN pills (for example: transport, closed pharmacies, higher cost of pills, etc)?**									**<0.001**
Yes	61	15.4	61	32.4	0	0	0	0	
No	330	83.3	124	66.0	87	97.8	119	100	
Unsure/Don’t Know	3	0.8	3	1.6	0	0	0	0	
Missing/not asked	2	0.5	0	0	2	2.2	0	0	
**Did COVID-19/lockdown affect your ability or comfort to TAKE your pills/WHEN you took your pills?**									**0.002**
Yes	17	4.3	16	8.5	1	1.1	0	0	
No	378	95.5	171	91.0	88	98.9	119	100	
Unsure/Don’t Know	1	0.3	1	0.5	0	0	0	0	
**Did COVID-19/lockdown affect your ability or comfort in seeking medical care during or after your abortion?**									**<0.001**
Yes	23	5.8	23	12.2	0	0	0	0	
No	373	94.2	165	87.8	89	100	119	100	
**Have you or anyone in your household lost wages since March 1**^**st**^ **as a direct or indirect result of COVID-19 and/or the lockdown? Select all that apply.**									**0.001**
Yes, I have lost wages	226	57.1	122	64.9	53	59.6	51	42.9	
Yes, someone in my household has lost wages	242	61.1	133	70.7	57	64	52	43.7	
No one in my household	82	20.7	9	4.8	19	21.3	54	45.4	
Not sure	1	0.3	1	0.5	0	0	0	0	

### Self-managed abortion outcomes

At last follow-up, the majority (n = 766/768, 99.7%) of participants who reported an outcome reported a complete abortion, most without the need for surgical intervention (Table 4). Only two participants (0.2%) reported an incomplete abortion, or uncertainty about abortion completion. Missingness for self-managed abortion outcomes was highest in the third phase of the pandemic and was due to loss to follow-up among participants in the ASQ study (the ASQ study protocol implemented fewer follow-up contacts than did the SAFE study, and did not prioritize abortion completion as an outcome). If we assume that all missing values were incomplete abortions, then the conservative estimate of abortion completion is 94.9%.

**Table 4:** Abortion completion following self-managed medication abortion among people who contacted a safe abortion accompaniment group in Lagos, Nigeria in 2019–2020.

	Overall		COVID-19 lockdown phases implemented in Nigeria								
			Pre COVID: Oct 2019-Mar 2020		Phase 1: Mar 31-June 1		Phase 2: June 2-Sept 3		Phase 3: Sept 4—Dec 31		Chi2 P-value
	*n = 807*		*n = 333*		*n = 266*		*n = 89*		*n = 119*		
**Self-managed abortion outcome**	n	%	n	%	n	%	n	%	n	%	
Complete abortion	766	99.7	330	99.4	265	100	81	100	90	100	0.45
*Complete without surgical intervention*	*593*	*77*.*2*	*329*	*99*.*1*	*264*	*99*.*6*	*0*	*0*	*0*	*0*	
*Complete with surgical intervention*	*2*	*0*.*3*	*1*	*0*.*3*	*1*	*0*.*4*	*0*	*0*	*0*	*0*	
*Complete*, *unsure of surgical intervention*	*171*	*22*.*3*	*0*	*0*	*0*	*0*	*81*	*100*	*90*	*100*	
Unsure/not complete	2	0.3	2	0.6	0	0	0	0	0	0	
*Missing*	*39*	*--*	*1*	*--*	*1*	*--*	*8*	*--*	29	--	

## Discussion

Findings from this pooled analysis describe the characteristics of people who self-managed an abortion with accompaniment group support in Nigeria before and during the COVID-19 pandemic and highlight the burdens people faced across the various lockdown phases, as well as benefits of self-managing at home. Despite the barriers experienced, the vast majority of participants were able to successfully complete their abortions outside the formal health system, in a place of their choosing (usually at home), with the virtual assistance of a safe abortion hotline/accompaniment group.

These findings highlight the impact that the pandemic had on people’s ability to obtain abortion care at each step of the abortion process–from obtaining a pregnancy test to confirm their pregnancy, to seeking health care during or after their self-managed abortion. These challenges undermined people’s ability to exercise their reproductive autonomy, and underscore the need for contraception, abortion, and other sexual and reproductive health services to be protected as essential health care services in any future pandemics.

At the same time, the findings emphasize the essential role of accompaniment groups. During a period when clinical abortion care became even more heavily restricted, the accompaniment model of counseling in a flexible, telehealth format empowered participants to safely and effectively manage their own abortion without needing to visit a clinic, and consequently with less need to arrange childcare or other logistical steps required to travel for care. The health care system in Nigeria was overburdened and strained, and national calls to reserve scarce health sector resources for those infected with COVID-19 all made accessing essential family planning care within the formal health system more challenging. The safe abortion accompaniment/hotline model filled a gap in supportive care that acknowledged the challenging unique circumstances of each person’s life, and enabled them to overcome these barriers and obtain high-quality, safe, and effective abortion care. Accompaniment groups provide an example of a person-centered model of care that was able to nimbly respond to the pandemic and ensure that their provision of essential services continued. Other service delivery organizations may be able to learn from these groups as they seek to adapt their services to center the needs and preferences of those they serve.

As with all research, this study had limitations. The COVID-19 defined time periods compared in these analyses cover different, non-overlapping periods of the year during which call volumes to the hotline varied, and thus some portion of the differences detected could reflect seasonal variation due to weather or school calendars, rather than differences due to the COVID-19 pandemic. In addition, we pooled data from two independent studies with different primary outcomes, objectives, and time points and thus it is possible that the combination of these datasets introduces measurement error for which we have not accounted. However, because the studies were conducted among nearly the same population of eligible people, and data were collected by the same study coordinators, these concerns are not severe. We were unable to reach all participants for follow-up, and thus cannot know how experiences or outcomes differed, if at all, for the participants lost to follow-up. Further, it is possible that the burdens imposed by the pandemic may have differed for those people who were not aware or, or unable to contact the safe abortion hotline, and thus were not enrolled in the study. However, these limitations are balanced by the strengths of our large sample size, and by the prospective, systematic design of data collection over each phase of Nigeria’s COVID-19 related lockdowns. This analysis leveraged data from two robust, prospective studies with unique datasets, and contributes detailed data on self-managed abortion experiences of Nigerians as they relate to the pandemic.

## Conclusions

In conclusion, results from this study underscore the challenges people faced during the COVID-19 pandemic in self-managing their abortions, and also the essential role that a safe abortion hotline played in expanding access to safe abortion during a time when the formal healthcare system was less accessible and higher-risk. Future research should explore how best to expand this model of care. As the global family planning community reflects on lessons learned from the COVID-19 pandemic, safe abortion hotlines and accompaniment groups stand out as a proactive model of abortion care that should be invested in and expanded to ensure that access to essential abortion care is not disrupted, even in future pandemics [23].
